# Supplementary material for: Functionalized Biochars as Supports for Ru/C Catalysts: Tunable and Efficient Materials for γ-Valerolactone Production
Source: Nanomaterials (Basel). 2023 Mar 22;13(6):1129. doi: 10.3390/nano13061129 (PMC10051761; doi:10.3390/nano13061129)
Supplement: Supplementary file 1 [file nanomaterials-13-01129-s001.zip › nanomaterials-2278718-supplementary.docx]

Supporting Information

Functionalized Biochars as Supports for Ru/C Catalysts:
Tunable and Efficient Materials for γ-Valerolactone Production

Charf Eddine Bounoukta ^1,2^, Cristina Megías-Sayago ^1^, Juan Carlos Navarro ^1^, Fatima Ammari ^2^,
Svetlana Ivanova ^1,^*, Miguel Ángel Centeno ^1^ and Jose Antonio Odriozola ^1^

^1^ Departamento de Química Inorgánica e Instituto de Ciencia de Materiales de Sevilla,
Centro Mixto CSIC-Universidad de Sevilla, 41092 Sevilla, Spain; charfeddinebounoukta@gmail.com (C.E.B.); cmegias@us.es (C.M.-S.); juancarlos.navarrodemiguel@kaust.edu.sa (J.C.N.);
centeno@icmse.csic.es (M.A.C.); odrio@us.es (J.A.O.)

^2^ Laboratoire de Génie des Procédés Chimiques-LGPC, Département de Génie des Procéés,
Faculté de Technologie, Université FERHAT ABBAS SETIF-1, Setif 19000, Algeria; [ammarifatima@yahoo.fr](mailto:ammarifatima@yahoo.fr) (F.A.)

* Correspondence: [svetlana@icmse.csic.es](mailto:svetlana@icmse.csic.es), sivanova@us.es

**Table of contents**

**Figure S1.** XRD patterns of the prepared catalysts.

**Figure S2.** Raman spectra of the prepared biochars.

**Figure S1.** XRD patterns of the prepared catalysts.

**Figure S2.** Raman spectra of the prepared biochars.
